# Alcohol in Greenland 1951–2010: consumption, mortality, prices

**DOI:** 10.3402/ijch.v71i0.18444

**Published:** 2012-12-17

**Authors:** Hans Aage

**Affiliations:** Department of Social Science, Roskilde University, Roskilde, Denmark

**Keywords:** alcohol, Greenland, alcohol policy, purchasing power, consumption, mortality

## Abstract

**Background:**

Fluctuations in alcohol consumption in Greenland have been extreme since alcohol became available to the Greenland Inuit in the 1950s, increasing from low levels in the 1950s to very high levels in the 1980s – about twice as high as alcohol consumption in Denmark. Since then, consumption has declined, and current consumption is slightly below alcohol consumption in Denmark, while alcohol prices are far above Danish prices.

**Objective:**

Description of historical trends and possible causal connections of alcohol prices, alcohol consumption and alcohol-related mortality in Greenland 1951–2010 as a background for the evaluation of the impact of various types of policy.

**Design:**

Time series for Greenland 1951–2010 for alcohol prices, consumption and mortality are compiled, and variation and correlations are discussed in relation to various policies aimed at limiting alcohol consumption. Corresponding time series for Denmark 1906–2010 are presented for comparison.

**Results:**

The trends in alcohol prices and consumption followed each other rather closely until the 1990s in Greenland and the 1980s in Denmark. At this time, consumption stabilised while prices decreased further, but the effect of prices upon consumption is strong, also in recent years. A trend in Greenlandic mortality similar to consumption is discernible, but not significant. Among alcohol-related deaths cirrhosis of the liver is less prevalent whilst accidents are more prevalent than in Denmark.

**Conclusions:**

The effect of alcohol excise taxes and rationing upon consumption is evident. The stabilisation and subsequent decline in consumption since the mid-1990s, while alcohol prices decreased persistently, does not preclude continued effects of prices. On the contrary, price effects have been neutralised by other stronger causes. Whether these are government anti-alcohol campaigns or a cultural change is not clear.

Alcohol policy rests on a causal chain with 2 links: from policies to consumption, and from consumption to public health. The purpose of this study is to investigate the strength and stability of these 2 causal links by means of historical macro-data time series for Greenland 1951–2010 for alcohol consumption, alcohol prices and alcohol-related mortality and to discuss their significance for alcohol policy concerning prices as well as rationing measures and information campaigns. It is well known that alcohol policy has influenced the fluctuations in alcohol consumption in Greenland, which have been extreme since alcohol became available to the Greenland Inuit in the 1950s, increasing from low levels in the 1950s to very high levels in the 1980s – about twice as high as alcohol consumption in Denmark. Since the 1980s, consumption has declined with current consumption slightly below alcohol consumption in Denmark, while alcohol prices in Greenland are far above Danish prices. Health problems and social problems related to alcohol consumption are also well known (1). However, no detailed, long-term studies exist.

Alcohol habits in Greenland differ from those in Scandinavian and European countries (2, 3), and the pattern of alcohol-related causes of death also differs. Cirrhosis of the liver is less prevalent whilst alcohol psychosis, alcoholism and alcohol poisoning are more prevalent than in Denmark (4). Externally caused deaths, which are partly alcohol related, are more numerous than in Denmark. For comparison, corresponding time series for Denmark 1906–2010 are presented.

## Material and methods

Time series for Greenland 1951–2010 are compiled from a variety of official statistical sources and special investigations and adjusted in order to improve consistency so that variations over time and correlations between time series can be evaluated. Time series for Denmark 1906–2010 are presented for comparison.

### Alcohol consumption

Alcohol consumption in Greenland is well documented since 1975 by time series for consumption in litres of pure alcohol per inhabitant above 14 years, published by Statistics Greenland (5) and updated regularly. From 1975 back to the early 1950s, when alcohol became available in the shops for the Greenland Inuit, reliable data also exist due to 2 detailed and careful investigations (6, 7) initiated by Danish authorities, who were concerned about mounting social and health problems because of high levels of consumption – from 1960 sharply increasing levels.

### Alcohol mortality

Considered to be the most important alcohol-related causes of death, the following ICD codes suggested by the Danish Health and Medicines Authority (8) are included:

**Table:** 

ICD 8, 1968–93	ICD 10, 1994-	
291	F10	Alcohol psychosis
303	F10	Alcoholism
E860, N979-980	T51, X45, X65, Y15	Alcohol poisoning
571	K70, K74	Cirrhosis of the liver
577	K85, K86	Pancreatitis

The following quantitatively less important ICD 10 codes are included for the period after 1994:

**Table:** 

	G31.2	Degenerative changes of the nervous system caused by alcohol
	G62.1	Polyneuropathia alcoholica
	K29.2	Gastritis alcoholica
	I42.6	Cardiomyopathia alcoholica

Data for 1983–2009 were obtained from the files of the register of deaths of the Danish Health and Medicines Authority, except for 1994 where the data are missing. Data for 1968–1987 were obtained from the files of the National Board of Health of Greenland. For the years 1983–87, there is accordance between these 2 sources. Prior to 1968, registration of causes of death was incomplete in Greenland, as the Danish law of 1871 on inspection of corpses by a medical officer was not implemented in Greenland, and death certificates were not generally issued in Greenland until 1967. However, it is possible – by means of summary statistics published by the then Danish Ministry for Greenland, the Danish Board of Health and Statistics Denmark – to compile a time series back to 1951 for the number of deaths caused by diseases of the liver, including cirrhosis of the liver and several other non-alcohol-related diseases. However, the numbers are very small, and in 11 out of 17 years there are no reported cases at all.

External causes of death are to some – unknown – extent alcohol related, and a separate time series for these was obtained from official annual medical reports (9) and statistical yearbooks (10). They include (ICD 10 codes in parentheses) traffic accidents (V01-89), other accidents (V90-99, W00-X59, Y40-86, Y87.1-87.2, Y88-89), suicides (X60-84, Y87.0) and other external causes (X85-Y36).

### Alcohol prices and purchasing power

When investigating the strength and stability of the influence of alcohol prices upon alcohol consumption, corrections for changes in the general price level and in income per capita are obviously inescapable. In very rough figures, the general price level in Greenland has probably increased by a factor of 20 since 1951, real GDP (i.e. GDP at fixed prices) by a factor of 15 and nominal GDP (i.e. GDP at actual prices) by a factor of 300. In Denmark prices have increased by a factor of 60 since 1906, real GDP by a factor of 15 and nominal GDP by a factor of 900.

Therefore, assumptions are needed, and here the simplest and most plausible ones are used. The price of alcohol is divided by the general price level so that relative price changes for alcohol are considered independently of inflation. These “real prices of alcohol” are divided by real income per capita (GDP at factor cost at fixed prices). Thus, it is assumed that prices influence consumption because of changes in the ratio of real alcohol prices to real income per capita. For easy reading of figures, the reciprocal of this ratio is used and the resulting index is alcohol purchasing power, which indicates the amount of pure alcohol in litres, which may be bought by the GDP (at factor cost) per capita. A similar index was used by Thorsen (11).

(1) alcohol purchasing power =

$$\text{(2)}\frac{\text{(real GDP per capita)*(nominal price level)}}{\text{(nominal price of 1 litre of pure alcohol)}}=$$

$$\text{(3)}\frac{\text{(GDP at nominal prices)/(population)}}{\text{(alcohol consumption at nominal prices)/(litres of pure alcohol consumed)}}$$

The assumption that alcohol consumption is a function of alcohol purchasing power implies that the effects of prices and income are reciprocal so that the elasticities of consumption with respect to prices and with respect to income are assumed numerically identical (elasticity is the ratio between the percentage increase in consumption and the percentage increase in price or income, respectively). The fraction (3) demonstrates how the index is computed. The computation of 1 single price index for the various types of alcohol in the denominator of fraction (3) corresponds to an index with annually changing weights (Paasche's price index). Therefore, consumer's choice between various types of alcoholic beverages influences the index. The price of alcohol can be considered exogenous in the sense that any correlation between prices and consumption is due to consumers’ reaction to prices and not due to adjustment of prices because of changes in consumers’ demand.

Full national account statistics are only recently being established in Greenland (12), but authoritative estimates of GDP since 1979 are provided by Statistics Greenland and by The Economic Council of Greenland. For 1947 and 1955–1960, figures for total income in money and in kind for the Greenlandic population were presented in a pioneering study by Boserup (13). For the remaining years, figures for money income and taxation from a variety of mainly government sources (especially 14, 6) were used with various methods of interpolation.

The volume of homebrewed beer, beer, wine and spirits consumed are available from the sources mentioned above in relation to alcohol consumption supplemented by (14) and other sources. Information on prices of various types of alcoholic beverages mainly exist as price indices in the detailed and reliable annual reports published by the then Danish Ministry of Greenland and by Den Kgl. Grønlandske Handel (The Royal Greenland Company), which possessed a monopoly on trade in Greenland until 1950 and was in charge of imports until 1986, when it was transferred to Greenland's Home Rule and reorganised. Absolute prices in DKK are available for 1951 and 1956–1959 only (10), and the time series for prices in DKK for beer, wine and spirits are based on these and various subsequent indices for relative prices, which are linked together; the frequently changing sizes of import duties in DKK are also taken into account. Prices for home brewed beer are computed from the prices of malt and sugar (6, 14). Since the 1950s prices have increased by a factor of 15–20, and a reliability check is obtained by comparing time series values for recent years to actual prices. The time series prices in 2010 of 16 DKK for beer (33 cl.), 102 DKK for wine (75 cl.) and 385 DKK for spirits (70 cl.) appear compatible with common shopping experiences.

### Danish time series

The Danish time series are compiled according to the same principles, and because of better data availability, it is possible to construct the series beginning in 1906, although not without using various partly arbitrary assumptions, as classification principles and practices have changed considerably during this long period. An important difference is, that in order to improve consistency of the Danish time series for alcohol-related deaths from natural causes, only alcohol psychosis, alcohol poisoning and cirrhosis of the liver are included, and not pancreatitis (ICD 10 codes K85, K86, approximately 10% of alcohol-related deaths from natural causes) and other quantitatively less important diagnoses. A full account of data compilation for Greenland and for Denmark is available from the author upon request.

## Results

### Alcohol consumption

**Fig. 1:**
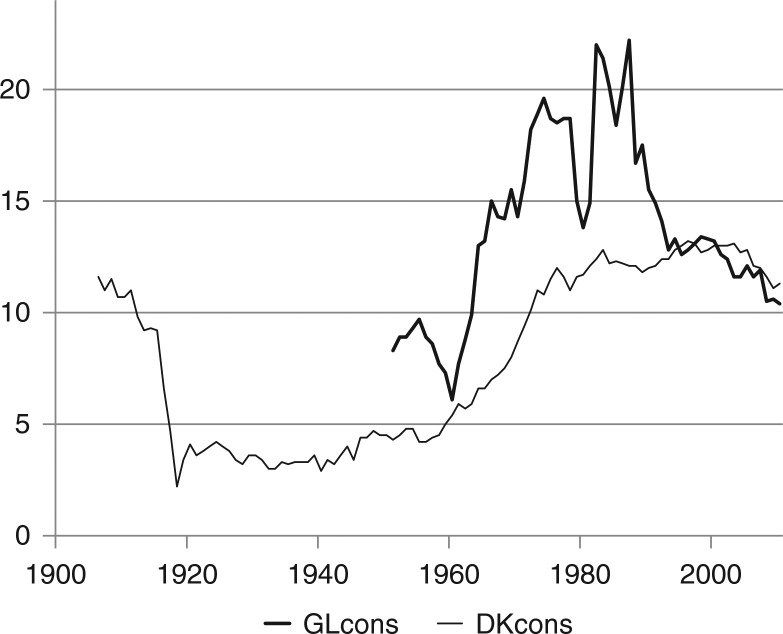
Alcohol consumption in Greenland (1951–2010) and Denmark (1906–2010). Note: annual consumption in litres pure alcohol per inhabitant above 14 years. GLcons: alcohol consumption in Greenland 1951–2010, DKcons: alcohol consumption in Denmark 1906–2010.

The recurrent dramatic fluctuations in alcohol consumption in Greenland and the periodic high levels by Danish and international standards are illustrated in Fig. 1, see Table I.

**Table I:** Alcohol in Greenland 1951–2010: consumption, mortality, prices

	Consumption	Cirrhosis hepatis	External causes	Alcohol mortality	Purchasing power		Consumption	Cirrhosis hepatis	External causes	Alcohol mortality	Purchasing power
											
1951	8.3	0.0	317.3	–	160	1981	14.9	5.5	334.8	19.4	185
1952	8.9	0.0	268.0	–	117	1982	22.0	5.4	369.5	10.7	169
1953	8.9	0.0	291.3	–	97	1983	21.4	15.7	295.4	54.9	176
1954	9.3	0.0	359.1	–	95	1984	20.1	7.7	370.9	38.7	170
1955	9.7	0.0	333.4	–	83	1985	18.4	10.0	298.7	37.7	153
1956	8.9	12.4	471.8	–	71	1986	20.1	5.0	300.9	29.8	157
1957	8.6	12.4	487.9	–	78	1987	22.2	2.5	328.2	27.1	175
1958	7.7	12.4	310.5	–	71	1988	16.7	7.3	338.0	41.3	157
1959	7.3	0.0	550.5	–	66	1989	17.5	14.5	311.9	36.3	149
1960	6.1	0.0	312.2	–	65	1990	15.5	2.4	314.5	33.9	141
1961	7.7	0.0	422.8	–	78	1991	14.9	7.3	292.2	36.5	137
1962	8.8	0.0	427.4	–	74	1992	14.1	4.3	248.6	29.5	122
1963	9.9	5.0	442.8	–	89	1993	12.8	7.5	203.9	17.4	121
1964	13.0	0.0	513.4	–	94	1994	13.3	–	221.1	–	123
1965	13.2	14.4	421.5	–	90	1995	12.6	2.5	262.7	32.2	131
1966	15.0	0.0	373.7	–	102	1996	12.8	0.0	227.7	12.4	134
1967	14.3	8.7	366.8	–	94	1997	13.1	4.9	283.8	17.3	137
1968	14.2	4.2	350.1	12.5	106	1998	13.4	9.8	265.4	14.7	147
1969	15.5	7.9	268.7	19.8	112	1999	13.3	4.9	245.5	29.5	150
1970	14.3	11.6	294.1	34.8	102	2000	13.2	9.8	227.4	48.9	159
1971	15.9	7.6	334.4	26.6	109	2001	12.6	0.0	250.9	31.7	174
1972	18.2	10.9	300.4	25.3	114	2002	12.4	7.2	242.8	28.8	178
1973	18.9	3.5	430.4	17.5	108	2003	11.6	9.6	210.3	40.6	175
1974	19.6	10.8	259.8	23.3	118	2004	11.6	11.8	233.7	35.4	187
1975	18.7	6.5	175.5	16.3	112	2005	12.1	2.3	243.9	44.5	190
1976	18.5	9.5	240.0	25.3	121	2006	11.6	9.3	228.9	32.7	202
1977	18.7	9.2	245.7	15.4	131	2007	11.9	4.7	191.2	39.6	212
1978	18.7	3.0	240.5	15.2	146	2008	10.5	0.0	157.9	7.0	187
1979	15.0	17.8	308.6	20.8	144	2009	10.6	4.6	169.0	25.5	193
1980	13.8	5.7	324.8	17.2	156	2010	10.4	–	208.6	–	193

Sources and notes: See text; full details of compilation of data for Greenland as well as for Denmark are available from the author on request.

Consumption: litres of pure alcohol annually per inhabitant above 14 years.

Mortality: deaths per 100,000 inhabitants above 14 years by cirrhosis of the liver (1968–2009, all liver diseases 1951–1967), by external causes (1951–2010) and by all alcohol-related causes (1968–2009, including cirrhosis of the liver, but not external causes).

Purchasing power: litres of pure alcohol, which can be bought by the GDP (at factor cost) per capita.

In the years prior to rationing being finally removed in December 1954, access to purchasing alcohol became gradually less restrictive for the Greenland Inuit. In 1953, Greenland was transferred from colonial status to part of Denmark as a county. The high level of alcohol consumption in Greenland as compared to Danish consumption at that time is partly explained by a relatively high consumption among the Danish population in Greenland, which in 1970 consumed approximately 50% more alcohol per capita than the Greenland Inuit; in 1970 25% of the population was born in Denmark, and in 2008 11% (7).

But first of all the explanation is widespread production and consumption of homebrewed beer among the Greenland Inuit in the 1950s. In 1951, 78% of total consumption was homebrewed beer, brewed by combining on average 1 kg of malt with 1.5 kg of sugar, which would produce 29 litres of beer with an average volume alcohol content of 3.9% (6). Home production declined rapidly after 1951, and it has been negligible since 1972. It was replaced by bottled beer and also by spirits, the share of which reached 30% in the 1960s. After a temporary decline to 6.1 litres per inhabitant above 14 years in 1960, total consumption more than trebled to a peak of 22.2 litres in 1987, distributed among beer (71%), wine (18%) and spirits (11%). The percentage distribution is almost unchanged in recent years, and it differs from the Danish distribution (beer 45%, wine 40%, spirits 15%). However, consumption has been cut by more than half to 10.4 litres in 2010, so it is now almost back at the level of 1955 and slightly smaller than current consumption in Denmark, which is also decreasing.

### Alcohol mortality

**Fig. 2:**
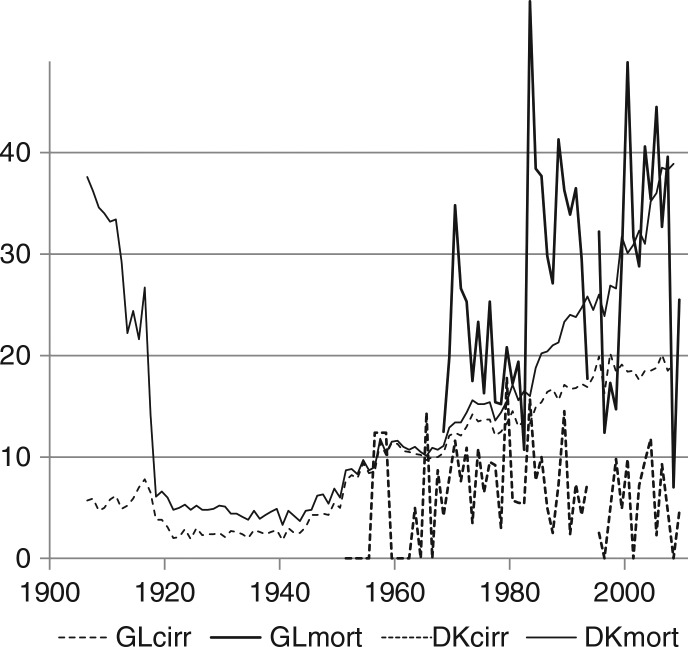
Deaths caused by alcohol generally and by cirrhosis of the liver specifically, in Greenland (1968–2009 and 1951–2009, respectively) and in Denmark (1906–2008). Note: deaths per 100,000 inhabitants above 14 years caused by cirrhosis hepatis, GLcirr (Greenland 1968–2009, 1951–1967 all liver diseases), DKcirr (Denmark 1906–2008), and by alcohol generally, GLmort (Greenland 1968–2009), DKmort (Denmark 1906–2008), which include besides cirrhosis of the liver also alcohol psychosis and delirium tremens, alcoholism, alcohol poisoning and – for Greenland, but not for Denmark – pancreatitis and after 1993 also some additional quantitatively less important alcohol-related diagnoses, cf. text.

For the last 25 years, average alcohol mortality per 100,000 inhabitants above 14 years was 28.2 in Greenland (1983–2009) compared to 28.4 in Denmark (1983–2008). For mortality caused by cirrhosis of the liver, the figures were 7.0 and 18.3, respectively, reflecting the well-known – but less well understood – fact that the prevalence of liver diseases in the Greenland Inuit is less than in European populations with comparable levels of alcohol consumption (4). Of the total number of alcohol-related deaths from natural causes in the period 1968–2009, 24% were caused by cirrhosis of the liver in Greenland and 64% in Denmark, the remainder by alcohol psychosis, alcoholism and alcohol poisoning, see Fig. 2.

Absolute numbers are tiny and therefore highly volatile. Greenland's population size was 24,000 in 1951 increasing to 56,000 in 1990, when the size stabilised. For the period 1983–2009, the average absolute annual number of deaths caused by cirrhosis of the liver was 2.9 and the average absolute annual alcohol mortality was 12.0, see Table I. However, some unclear trends are probably discernible from Fig. 2: for deaths caused by cirrhosis of the liver an increase from the 1950s until the 1980s and thereafter a slightly lower level; for general alcohol mortality an increase in 1983 to a permanently higher level, but annual variations are large.

In Denmark, alcohol mortality has increased steadily since the 1950s and is now back at the level before the large upsurge of alcohol excise taxes during World War I. Simultaneously, the share of deaths caused by cirrhosis of the liver increased. However, the reason for most of the increase in alcohol mortality since the 1990s is due to traditional causes – alcohol psychosis, alcoholism and alcohol poisoning have returned, after having been virtually absent for most of a century.

**Fig. 3:**
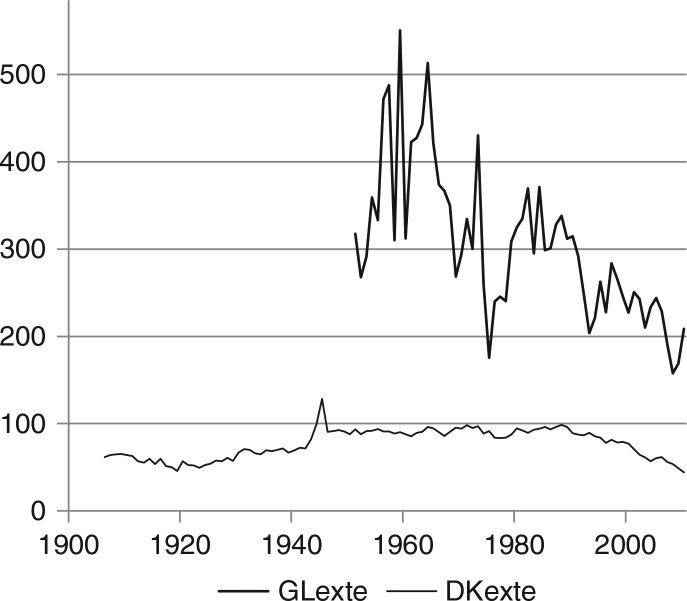
Deaths caused by external causes in Greenland (1951–2010) and in Denmark (1906–2010). Note: deaths per 100,000 inhabitants above 14 years caused by external causes (traffic accidents, other accidents, suicides and other external causes) in Greenland (GRexte) and Denmark (DKexte).

The causes of alcohol mortality included in Fig. 2 understate alcohol mortality, because alcohol-related injuries and accidents are omitted, the number of which is relatively high in Greenland. In 1959, the medical officer of health in Greenland declared: “Innumerable larger and smaller injuries, not infrequently the consequence of assaults and gross violence, and several fatally ending accidents are caused by intoxication. Consumption of alcohol is disquietingly excessive …” (9). Figure 3 shows the numbers of deaths due to external causes, traffic accidents, other accidents, suicides and other external causes, which are often related to alcohol (3), although the extent is unknown.

Of all the deaths in Greenland, approximately 20% are unnatural deaths, that is due to external causes, as compared to less than 5% in Denmark, and the composition also differs. In Greenland, there are very few traffic accidents, and other accidents and suicides each account for approximately half of the unnatural deaths. In Denmark, approximately 15% are traffic accidents, 25% suicides and 60% other accidents and other external causes. In Greenland, the number of unnatural deaths shows a long-term declining trend because of a decline of the number of accidents since the 1970s; the number of suicides was low in 2007–2009, but increased again in 2010, and there is no clear trend. In Denmark, the number of unnatural deaths has declined by 50% since the 1980s; the number of suicides and traffic accidents has both been halved, and the number of other accidents is also declining.

### Alcohol prices and purchasing power

**Fig. 4:**
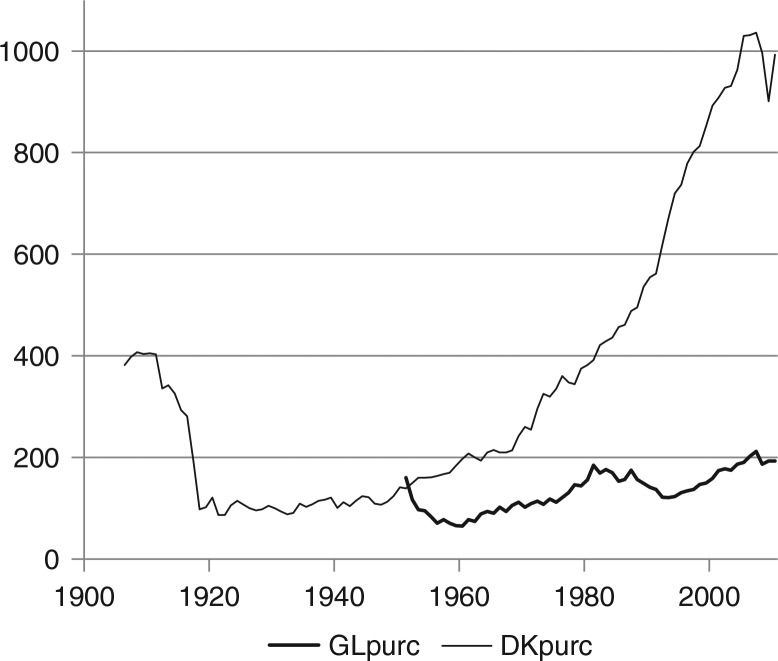
Alcohol purchasing power in Greenland (1951–2010) and Denmark (1906–2010). Note: alcohol purchasing power indicates the amount of pure alcohol in litres, which can be bought by the GDP per capita (at factor cost). GLpurc: alcohol purchasing power in Greenland 1951–2010. DKpurc: alcohol purchasing power in Denmark 1906–2010.

From 1960 to present day, alcohol purchasing power increased by a factor of 3 in Greenland as compared to a factor of 5 in Denmark, and alcohol purchasing power in Greenland is only 20% of the level in Denmark, see Fig. 4.

Although there is a whole range of prices for various types of alcohol, rough estimates would indicate that prices in Greenland as a percentage of Danish prices are 300% for beer, 250% for wine and 350% for spirits.

The comparison with Denmark is complicated because of the annual subsidy from the Danish government, approximately 3.5 milliard DKK or 30% of Greenland's GDP of 12 milliard DKK in 2010. This subsidy enters Greenland's economy through the government coffers, and it allows government spending on transfer payments (or lower taxes), which increase personal incomes, and on public consumption, which increases personal incomes as well as GDP directly. The subsidy also allows for a permanent import surplus in Greenland. Government consumption in Greenland is 50% of GDP as compared to 30% in Denmark (12), but relative to GDP plus an import surplus equal to the Danish subsidy, the share is less, approximately 40%. To trace the ramifications of the effects of the Danish subsidy in Greenland's economy requires a complete macroeconomic model and would be highly hypothetical (15, 16). As an approximate percentage of the corresponding Danish figures, GDP per capita in Greenland is approximately 75%, and without the subsidy it would be much lower. The subsidy also allows for an import surplus and increases the disposable amount to approximately 100%. Retail prices, which are on average 20% higher in Greenland than in Denmark, should also be taken into account. There is no obvious way of correcting for the effects of the Danish subsidy upon the alcohol purchasing power index.

It could be argued that personal disposable income (income less taxes plus government transfer payments) or private consumption would be preferable to GDP as a correction for general prosperity in society. However, this is not obvious as government consumption is also consumption and contributes to prosperity, even if it is decided upon collectively. If government consumption were removed from GDP in the index, purchasing power in Greenland would be even less compared to the Danish level. For all its shortcomings, GDP serves its purpose in relation to the index. The correction for general price and income increases is a necessary scaling of the index– of which the big jumps are mostly caused by changes in alcohol prices.

### Causal relations between prices, consumption and mortality

**Fig. 5:**
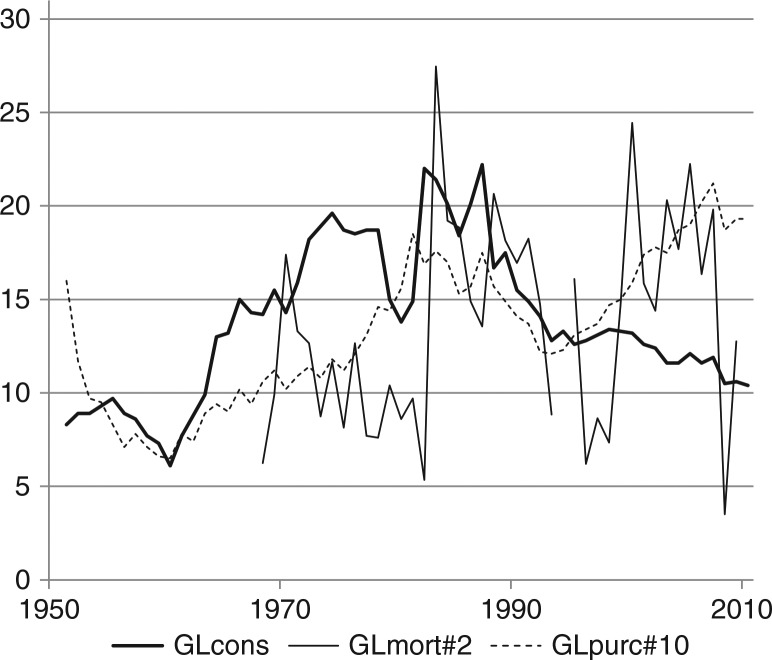
Alcohol consumption, mortality and purchasing power in Greenland (1951–2010). Note: time series have been scaled in order to illustrate possible correlations. GLcons: alcohol consumption, see Fig. 1 and Table I. GLmort#2: alcohol mortality divided by 2, see Fig. 2 and Table I. GLpurc#10: alcohol purchasing power divided by 10, see Fig. 3 and Table I.

**Fig. 6:**
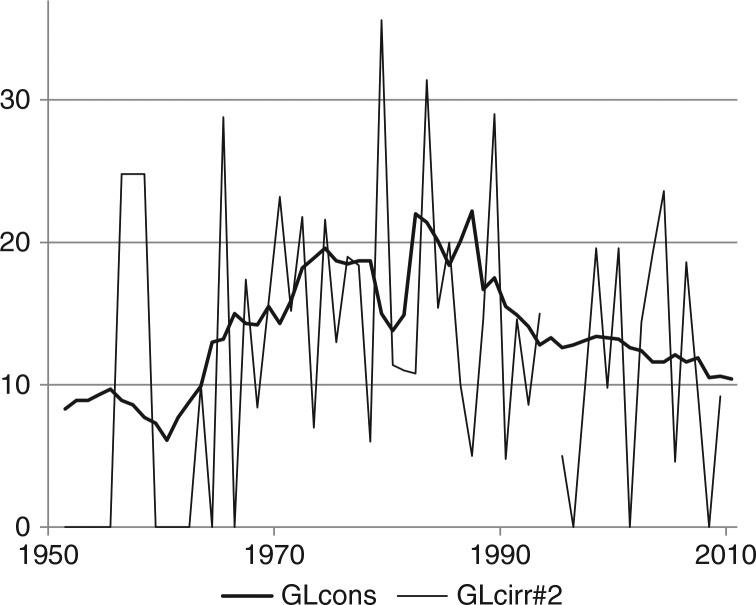
Alcohol consumption and mortality caused by cirrhosis of the liver in Greenland (1951–2009). Note: see Fig. 5; GLcons see Fig. 1 and Table I. GLcirr#2: mortality caused by cirrhosis of the liver divided by 2, see Fig. 2 and Table I. In the years 1951–67 all liver diseases are included.

**Fig. 7:**
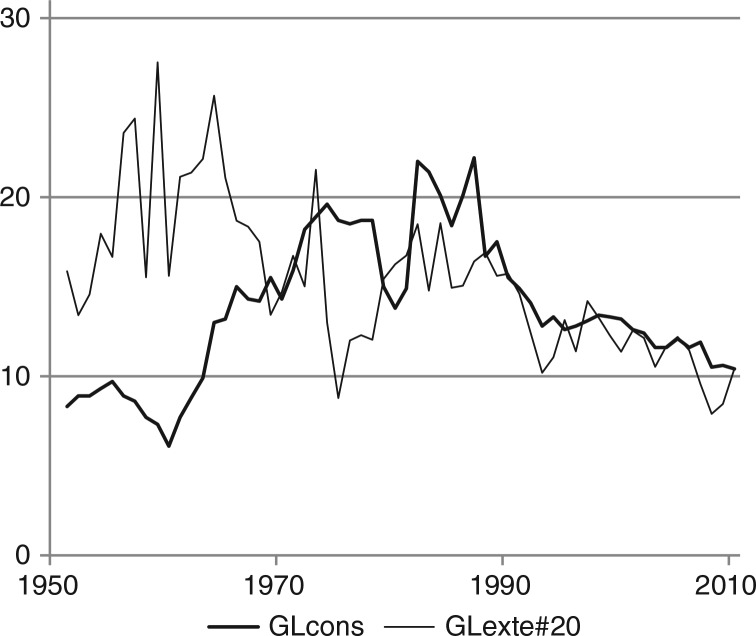
Alcohol consumption and mortality caused by external causes in Greenland (1951–2010). Note: see Fig. 5; GLcons see Fig. 1. GLexte#20: mortality caused by external causes divided by 20, see Fig. 3 and Table I.

The effects of the causal chain from prices and income to consumption and eventually to mortality are revealed in Figs. 5–7, where the 3 times series are displayed together, with no manipulations except that the measurement scales of purchasing power and mortality are adjusted in order to illustrate possible correlations.

The correlation between alcohol purchasing power and consumption is unmistakable from the mid-1950s to the early 1990s. From 1955 to 1994, the annual percentage increase in consumption is explained by annual percentage increase in purchasing power in the same year with a significant coefficient of 0.54 and a correlation of r=0.39. The coefficient is an estimate of the elasticity of consumption with respect to purchasing power. If a stable relationship could be assumed between the 2 variables, a constant elasticity probably is the most likely possibility; it is estimated from the first differences of the logarithms of the 2 variables. From 1995 to 2010, there is an almost significant (p=0.063) elasticity of 0.46 and a correlation of r=0.54. The elasticity estimates are of comparable magnitude, and the regressions indicate a causal effect of purchasing power upon consumption, even after 1995, where a downward trend in consumption is partly offset by increasing purchasing power.

The instances of marked deviation between changes in alcohol purchasing power and consumption must be explained in terms of changes in other alcohol policy instrument than duties and prices, first of all various forms of rationing (5, 3). The increase in consumption from 1951 to 1955, when purchasing power declined, is explained by the gradually less restrictive access to buying alcohol for the Greenland Inuit until general rationing was lifted in December 1954. As prices continued their increase, consumption also declined to a low in 1960 (6). From 1960 purchasing power increased again, more than doubling until 1979, while consumption trebled. A law concerning strong beverages from April 1973, which restricted opening hours for alcohol sale – subsequently frequently changed – and introduced local rationing, had little effect. Following a referendum in 1978, a ban on home production of alcohol and on imports of malt and hobs was established in August 1979 as well as a general rationing, according to which adults above 18 years of age received 72 *points* every month; 1 *point* allowed purchase of alcohol corresponding to the contents of a 33 cl. bottle of beer. Rationing gave rise to unintended activities including trade in *points*, the price of which could reach 25 DKK per *point* according to anecdotal information; the shop price of 1 bottle of beer was 5 DKK. Registered consumption dropped from 18.7 litres of alcohol per inhabitant above 14 years in 1978 to 13.8 litres in 1980, but immediately soared to 22.0 litres in 1982, when in March 1982 rationing was lifted except in some local areas. In the following years, alcohol excise taxes were gradually increased, and from a maximum of 22.2 litres in 1987 consumption began a persistent decline. In June 1992, alcohol taxes became progressive according to alcohol percentage of beverages; availability was liberalised in these years, especially for the new, weak beer with 3.6% alcohol, but local rationing was retained in some areas for various types of alcoholic beverages, and these rules as well as local regulations for opening hours were frequently changed and gradually became more restrictive. The decline in consumption continued despite an increase in purchasing power of 76% from a minimum in 1993 until 2007. However, when alcohol purchasing power was cut by 12% from 2007 to 2008, it induced a decrease in consumption, also by 12%, and since then consumption has stabilised on a relatively low level, lower than at any time since 1964, see Fig. 5. Similarly, there is a trend among school children towards less alcohol intake (17, 18).

Rates of mortality due to alcohol in general and cirrhosis of the liver specifically are computed from very small and volatile absolute numbers, but some correlation with consumption is discernible from Figs. 5 and 6. If the 2 last observations (for 2008 and 2009) are disregarded in Fig. 5, mortality seems to follow the downward trend of consumption in the 1980s and then increase despite the stabilisation of consumption, much like the increase of mortality in Denmark since the 1980s. Whether the low mortality rates in 2008 and 2009 are outliers or indicate a turn of the trend, remains to be seen.

If there is a stable relationship between consumption and mortality, it is reasonable to expect that absolute changes in mortality are affected by the absolute change in alcohol intake during preceding years. The statistical correlations between absolute increase in consumption over the preceding 5 and 10 years, respectively, and absolute annual increase in mortality (due to alcohol generally and to cirrhosis of the liver) are, however, very weak and far from significant, although mostly positive.

Despite very low absolute numbers, the trend in mortality caused by cirrhosis of the liver seems to follow consumption, and it seems to stabilise concurrently with the stabilisation of consumption, see Fig. 6. Again, this resembles the development in Denmark, where, however, mortality due to cirrhosis of the liver continued its increase a decade after the stabilisation of consumption and only became stable in the 1990s; the continued increase in alcohol mortality is due to the traditional causes, alcohol psychosis, alcoholism and alcohol poisoning.

Clearly, alcohol intake increases the probability of injuries and accidents at a micro level, but it is not obvious that this causal effect is also reflected in macro-data as in Fig. 7.

The similarity of the trends since the 1970 most likely reflects a causal relationship, but it seems more likely, that mortality due to external causes displays a long-term declining trend, which to some extent coincides with the trend in consumption since 1980. The declining trend might have other causes not related to alcohol including improvements of the social situation and gradually less hazardous working conditions. If there were a correlation between absolute annual changes in consumption and in externally caused mortality, respectively, it would be a stronger indication of a causal effect. Before 1980, the correlation between first differences is negligible although positive. After 1980, there is a positive correlation (r=0.22), but it is far from significant. Therefore, the data only faintly support a causal relationship between alcohol consumption and the number of deaths due to external causes.

**Fig. 8:**
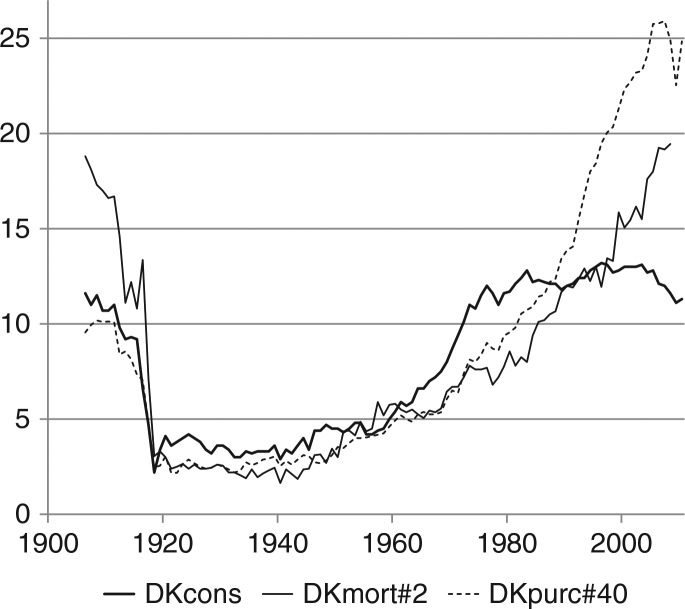
Alcohol consumption, mortality and purchasing power in Denmark (1906–2010). Note: see Fig. 5 and Table I. DKcons: alcohol consumption, see Fig. 1. DKmort#2: alcohol mortality divided by 2, see Fig. 2. DKpurc#40: alcohol purchasing power divided by 40, see Fig. 4.

The 3 time series for Denmark 1906–2010 for alcohol prices, consumption and mortality display a long-term, extremely close correlation, quite exceptional for the social sciences, namely between 1906 and the mid-1980s, see Fig. 8.

It looks like a law of nature, but it is not. The mechanisms, the biological as well as the social, were not the same during these 75 years. When the price of spirits, “snaps”, was increased by a factor of 10 in April 1917 – in order to secure sufficient provisions of grain and potatoes for feeding the population – alcohol consumption was a male, lower class phenomenon, and the prevailing alcohol-related diseases were alcohol psychosis, alcoholism and alcohol poisoning (19, 20). When consumption increased again after 40 years of low levels of purchasing power, consumption and mortality, it was part of a general boom in prosperity. Consumption became much more evenly distributed over time, among social groups and among men and women. Consequently, the dominant alcohol-related diseases, alcohol psychosis and poisoning, were replaced by diseases caused by more moderate, long-term consumption, especially cirrhosis of the liver, and women's share of all alcohol-related deaths from natural causes increased from 10–20% to 30–50%.

Therefore, statistical analysis is not appropriate, neither is it necessary, even if regression analysis gives a nice fit. From 1906 until 1982, the annual percentage increase in consumption is explained by annual percentage increases in purchasing power in the same year with an estimated elasticity of consumption with respect to purchasing power of 0.84 and a correlation of r=0.74. Annual absolute increase in mortality from 1906 until 1983 is explained by an absolute increase in consumption over the preceding 5 years with a coefficient of 0.50 and a correlation of r*=*0.42. These correlations are highly significant, but should be regarded as spurious, as the underlying causal structures are not stable.

For the period post 1983, more stable causal relationships could possibly be presumed. After 1983, the relationship between purchasing power and consumption is still significant, but weaker (r=0.55, elasticity=0.32). After 1984, there is a weak negative and insignificant correlation between consumption and mortality. Alcohol mortality continued its increase presumably because of its more long-term nature, and mortality due to cirrhosis of the liver increased. However, since the 1990s most of the increase in alcohol mortality is due to a return of traditional causes, alcohol psychosis, alcoholism and alcohol poisoning, and the number of deaths caused by cirrhosis of the liver seems to stagnate, see Figs. 2 and 8.

As per information in Fig. 8, the common trends came to an end in the late 1970s, when alcohol consumption levelled off, stabilised and eventually began a modest decline, while alcohol purchasing power almost trebled during the following 30 years. However, prices still seem to matter and the elasticity is significant; an example is the 13% drop in purchasing power from 2007 to 2009, which was followed by an 8% decline in consumption. In Sweden, there were significant price elasticities (without corrections for income changes) for beer, wine and spirits with numerical values of 0.60–1.00 (decreasing elasticity for beer) for the years 1984–2003, when consumption changed considerably, increasing for beer and wine and decreasing for spirits (21). In Norway, Sweden and Denmark, there was a strong positive relationship between changes in income (without corrections for price changes) and changes in alcohol consumption for the long time span from 1851 to 2002 (20). However, for Denmark during the period 1980–2005, there was no impact of income (without corrections for price changes) upon consumption, but the composition of consumption was sensitive to relative prices as can be demonstrated by advanced statistical methods, although the switch towards wine consumption, up from 20% to 40% in 1980, is explained by changing tastes and not by a change in relative prices (22).

A plausible interpretation of the diverging trends together with positive annual elasticities of consumption with respect to purchasing power observed in Greenland (Fig. 5) as well as in Denmark (Fig. 8) is that the impact of purchasing power is first of all due to price changes, and that a strong external downward trend in consumption is in part countervailed by purchasing power increases so that the decline of consumption would have been larger if purchasing power had not increased.

## Discussion

The data used are macro-indicators and therefore highly simplified. The purchasing power indicator presupposes that the effects upon alcohol consumption of prices and of income are exactly reciprocal, which could well be oversimplified. However vague, the time series disclose conspicuous trends. Alcohol purchasing power in Greenland is very much below the Danish level, approximately 80% below, but consumption is about the same after a decline from levels far above Danish consumption in the 1970s and 1980s. The influence of purchasing power upon consumption is unmistakable in Greenland as in Denmark, although this effect is compounded with various measures of quantitative rationing in Greenland. Since the mid-1990s purchasing power has increased in Greenland, while consumption has decreased, but nevertheless there is a positive, almost significant correlation also for the years 1995–2010.

There are no significant effects of alcohol consumption upon alcohol mortality as measured by these macro-data, although weak correlations are discernible. In Denmark this effect is evident up until the 1980s. If the high incidence of unnatural deaths in Greenland is ignored, which are partly alcohol related, the level of alcohol-related mortality in Greenland is comparable to Danish mortality, but cirrhosis of the liver is less prevalent. The data are subject to a number of limitations:

- First of all, the number of alcohol-related deaths from natural causes in absolute terms is small and volatile.
- Furthermore, total consumption of pure alcohol ignores differences and changes in consumption patterns. Alcohol intake is more concentrated in Greenland than in Denmark (2), but consumption has become more evenly distributed over time and across groups of the population, social and geographical. Thus, gender differences are diminishing in Denmark; there are no data for alcohol consumption of women and men separately in Greenland (23), but consumption for women and men is converging towards a common pattern (1).
- The mortality time series are crude indicators of alcohol-related deaths, and of course even more so of general health and social effects of alcohol consumption (6, 7, 24, 25). Specifically concerning external causes of death, there is no possibility of distinguishing between those that are alcohol related, and those that are not.
- Mortality rates are not age adjusted except for the conventional adjustment of leaving out children below the age of 14. However, alcohol-related death mostly occurs above the age of 40, and the number of persons above the age of 40 as a percentage of the population above the age of 14 has changed in Greenland; it was 32% in 1980 and 55% in 2010. An attempt to adjust mortality rates for this ageing of the population would change the trend towards more decrease over time, but not the volatility and therefore not the lack of significant correlation between consumption and mortality.

Even if the recent purchasing power increase– in Greenland since the mid-1990s by 75%, and in Denmark since the early 1980s by 200% – took place simultaneously with stabilisation of alcohol consumption at a moderate level and a subsequent further decline, this does not preclude a continued impact of prices, and the elasticity of consumption with respect to purchasing power is still positive and significant in Denmark and almost significant in Greenland. This indicates that consumption is subject to a long-term decreasing trend, but still sensitive to purchasing power changes, first of all prices changes, as prices fluctuate more than income. The consumption decrease had presumably been stronger, if not increasing purchasing power had partially counteracted the trend.

The decreasing long-term trend in consumption must be explained by other forces than prices. Beginning in the 1990s government has initiated alcohol prevention campaigns in Greenland as well as in Denmark, including recommended maximum alcohol consumption limits and an annual “No alcohol week” since 1992 in Greenland. Does this demonstrate an efficiency of government campaigns comparable to the efficiency of excise taxes and quantitative rationing? Probably not. The levelling off of consumption in Denmark began in the early 1980s, more than a decade before government campaigns took off in the 1990s. And stagnating alcohol consumption seems to be a widespread trend in recent decades in most European countries (although not, for example, in Sweden (21)) and in the Americas, whereas consumption is increasing in Asia. In 2002 the EU annual average adult consumption of pure alcohol was 11 litres, well above the world average of approximately 5 litres, but down by 30% from over 15 litres in the mid-1970s (26). A possible interpretation is that the consumption decline and the campaigns might both be manifestations of a common underlying cause, namely a more fundamental, cultural turn of the tide.
